# Supplementary material for: LDPE Transformation by Exposure to Sequential Low-Pressure Plasma and TiO2/UV Photocatalysis
Source: Molecules. 2021 Apr 26;26(9):2513. doi: 10.3390/molecules26092513 (PMC8123516; doi:10.3390/molecules26092513)
Supplement: Supplementary file 1 [file molecules-26-02513-s001.zip › molecules-1103431-supplementary.pdf]

molecules

MDPI

LDPE Transformation by Exposure to Sequential Low-Pressure Plasma and TiO_2/UV Photocatalysis

Luis D. Gómez-Méndez [1,2], Luis C. Jiménez-Borrego [2], Alejandro Pérez-Flórez [3], Raúl A. Poutou-Piñales [4], Aura M. Pedroza-Rodríguez [1], Juan C. Salcedo-Reyes [2], Andrés Vargas [5], and Johan M. Bogoya [6]

Supplementary Material 1: *Direct Current low-pressure plasma (DC-LLP) - Assembly and operating conditions)*

The chamber was connected through a butterfly valve to a turbo vacuum pump (Pfeiffer ™). Vacuum pressure was monitored through Pirani sensors and a cold cathode. Coaxial electrodes were (made of conducting flat parallel 8 cm diameter disks (cathode: copper (Cu^{2+}) and aluminum (Al^{2+}), anode: copper (Cu^{2+})) were and placed vertically concerning the chamber with 5.6 cm between them. With the anode grounded, the voltage between the electrodes ranged between 500 and 1000 VDC. Clean LDPE sheets were placed in the anode that served as the LDPE substrate-holder. To generate the DC-LPP the chamber was cleaned performing a vacuum up to 9×10^{-5} mbar employing the butterfly valve with the turbo molecular system. Subsequently to dose the vacuum the butterfly valve was partially closed, and the vacuum was reduced to 4×10^{-4} mbar. Once this pressure was reached the dosing valve connecting with the chamber was opened before the previous injection of Ar-O_2 mix at the selected concentration. The pressure was adjusted between 2×10^{-3} and 2×10^{-2} mbar to obtain a DC - EGD plasma. The system could reach equilibrium and voltage was increased until plasma appeared with the minimum of electric current required (Figure S1)

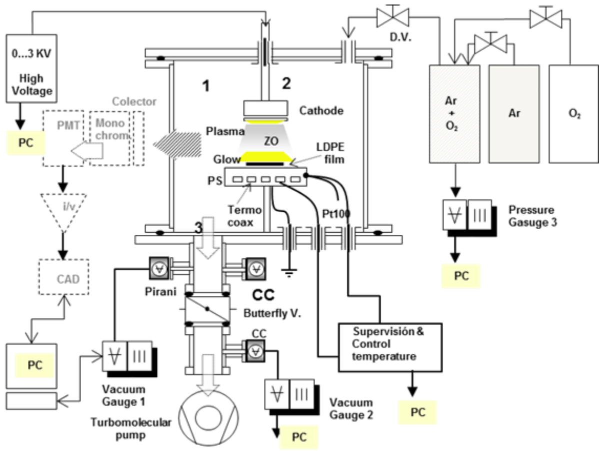

Figure S1. Schematic diagram of the plasma reactor employed in this study.

Supplementary Material 2: *Plasma discharge condition selection*

Initially, preliminary assays took place for each gas and then for the mix employing different voltages and pressures. For 100% O_2 (*v*/*v*) the following pressures were evaluated: 2.2×10^{-2}, 2.4×10^{-2}, 2.6×10^{-2}, 2.8×10^{-2}, and 3.0×10^{-2} mbar, with 1100, 1000, 900, 800, 700 and 600 VDC. The discharge time was set at 6 minutes. The pressures were the same for Ar; for this gas, voltages were 1300, 1200, 1100, 1000, 900, 800, 700, 600, and 500 V. For the Ar-O_2 1:1 mix the same pressures evaluated for gases in separate were considered, the same voltages were verified including a lower voltage at 400 V. The considerations to select parameter combinations (gas concentration, pressure, voltage, and time) were: (1) that they would not burn or damage LDPE sheets and (2) voltage conditions as a function of pressure would generate a stable plasma.

Table S1. Treatments employed for oxygen, argon, and argon-oxygen (50:50) mix.

Treatment	Type of Gas	Gas concentration (%)	Vacuum pressure (10^{-2} mbar)	Voltage (V)
1	O_2	100	2.4	1,000
2	O_2	100	2.8	700
3	O_2	100	2.8	800
4	O_2	100	3.0	600
5	O_2	100	3.0	700
1	Ar	100	2.2	1,000
2	Ar	100	2.2	1,100
3	Ar	100	2.2	1,300
4	Ar	100	2.4	800
5	Ar	100	2.4	900
6	Ar	100	2.6	900
7	Ar	100	2.6	1,000
8	Ar	100	2.8	800
9	Ar	100	2.8	900
10	Ar	100	3.0	500
11	Ar	100	3.0	600
12	Ar	100	3.0	700
13	Ar	100	3.0	800
1	Ar/O_2	50-50	2.2	1,000
2	Ar/O_2	50-50	2.2	1,200
3	Ar/O_2	50-50	2.4	800
4	Ar/O_2	50-50	2.4	900
5	Ar/O_2	50-50	2.4	1,000
6	Ar/O_2	50-50	2.6	700
7	Ar/O_2	50-50	2.6	800
8	Ar/O_2	50-50	2.6	900
9	Ar/O_2	50-50	2.8	500
10	Ar/O_2	50-50	2.8	600
11	Ar/O_2	50-50	2.8	700
12	Ar/O_2	50-50	3.0	400
13	Ar/O_2	50-50	3.0	500
14	Ar/O_2	50-50	3.0	600

Table S2: ANOVA results for Ar, O_2 and Ar-O_2 (50:50) mix.

**Final Angle (°) 100 % Argon**					
Descriptions	**SS**	**dF**	**MS**	**F Value**	**Proba > f**
Treatment	1431	11	130.1	2.963	**0.0126**
Residual	1054	24	43.91		
R square	0.5759				
Total	2485.57	35			
**Final Angle (°) 100 % Oxygen**					
Descriptions	**SS**	**dF**	**MS**	**F Value**	**Proba > f**
Treatment	67.92	3	22.64	1.035	**0.4275**
Residual	175	8	21.87		
R square	0.2796				
Total	243.19	11			
**Final Angle (°) 50% Oxygen & 50% Argon**					
Descriptions	**SS**	**dF**	**MS**	**F Value**	**Proba > f**
Treatment	1024	11	93.13	6.099	**0.0001**
Residual	366.5	24	15.27		
R square	0.7365				
Total 1391.23	35				
**Tukey test**					
Descriptions	**SS**	**dF**	**MS**	**F Value**	**Proba >f**
Treatment	300.3	5	60.07	4.623	0.0139
Residual	155.9	12	12.99		
R square	0.6583				
Total	456.85	17			

Supplementary Material 3: *Static contact angle*

In this work a spherical cap was assumed, where gravitational effects were negligible [66]. The profile of a sessile droplet (Figure S2) was defined by (Equation (1):

$$h(x) = R\sqrt{1 - \left(\frac{x}{R}\right)^2} - d$$

where: R is the spherical cap radius. According to the geometry from the ratio $\zeta = b/a$, where θ is defined as (Equation (2)):

$$sin(\theta) = \frac{2\zeta}{1 + \zeta^2}$$

The contact angle was determined by placing on LDPE's surface 50 μL of deionized water. Its mean value and its corresponding dispersion were calculated from three different positions observed on the sample through a JVC™ GZ-EX355 Everio video camera [31].

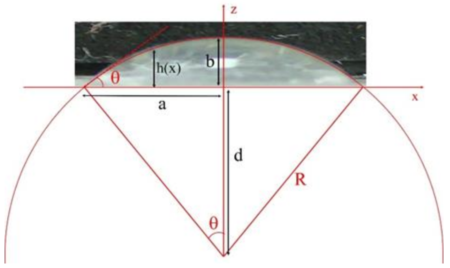

Figure S2. Profile of the spherical cap description for a sessile droplet. The image of a representative droplet before plasma treatment.

Table S3: SCA, roughness and LDPE's viscoelastic properties obtained during the 2^2-factorial design.

Static Contact Angle (%)				LDPE Final Weight of (%)			
Factor	p value	Contribution (%)	Stand. Effect	Factor	p value	Contribution (%)	Stand. Effect
Model	**< 0.0001**		+ 43.6	Model	**< 0.0001**		+ 3.54
A: pH	**< 0.0001**	45	+ 6.5	A: pH	**< 0.0001**	60	+ 0.47
B: TiO_2 Concentration	**< 0.0001**	40	+ 6.1	B: TiO_2 concentration	**0.0002**	38	+ 0.20
AB	0.6381	5	+ 0.5	AB	0.9002	2	+ $5.5x10^{-3}$
R^2	0.8223			R^2	0.8795		
CV	9.69			CV	5.46		
Adeq Precision	13.4			Adeq Preci-sion	15.630		
**Young modulus (Mpa)**				**Yield strength (Mpa)**			
Factor	p value	Contribution (%)	Stand. Effect	Factor	p value	Contribution (%)	Stand. Effect
Model	**< 0.0001**		+ 80.74	Model	**< 0.0001**		+ 4.35
A: pH	**< 0.0001**	33	- 11.54	A: pH	**< 0.0001**	70	- 1.02
B: TiO_2 concentration	**< 0.0001**	33	- 23.70	B: TiO_2 concentration	**0.0268**	20	- 0.48
AB	**< 0.0001**	33	- 24.85	AB	**0.0007**	30	- 0.82
R^2	0.9800			R^2	0.8100		
CV	6.65			CV	18.85		
Adeq Precision	40.423			Adeq Preci-sion	9.507		

Table S4: Response variables obtained during factorial design 2^2 (300 h)

	pH	TiO_2 (gL^{-1})	SCA (°)	Weight (mg)	Young's modulus (MPa)	Yield strength (MPa)
T1	4.5 ± 0.1	1.0 ± 0.1	35 ± 5 a	3.4 ± 0.5 a	92 ± 2	5 ± 2
T2	9.0 ± 0.1	1.0 ± 0.1	48 ± 6	3.8 ± 0.4	117 ± 21 a	4 ± 1
T3	4.5 ± 0.1	10.0 ± 0.1	49 ± 8	3.6 ± 0.6 a	94 ± 4	5 ± 2
T4	9.0 ± 0.1	10.0 ± 0.1	51 ± 7	4.2 ± 0.4	21 ± 1	2 ± 1a
PhC	ND	ND	61 ± 9	4.2 ± 0.3	48 ± 4	4 ± 1
Pc	ND	ND	51 ± 7	4.9 ± 0.1	41 ± 6	11 ± 2

All treatments: (PEBD + plasma +UV+ TiO_2)
PhC: Photolysis control (PEBD + plasma + UV)
Pc: Plasma control (PEBD +plasma)
ND: No data
